# Mental Health as a Key Mediator for Outcomes in Postsecondary Education, Employment, and Everyday Living in Autistic Adults

**DOI:** 10.1089/aut.2024.0122

**Published:** 2025-08-11

**Authors:** Mikle South, So Yeon Park, Molly Berman

**Affiliations:** ^1^ Department of Psychiatry and Behavioral Sciences, Emory Autism Center, Emory University School of Medicine, Atlanta, Georgia, USA.; ^2^ Department of Psychology, Brigham Young University, Provo, Utah, USA.; ^3^ Department of Psychology, Marquette University, Milwaukee, Wisconsin, USA.

**Keywords:** autistic adults, postsecondary education, employment, adaptive living, mental health, anxiety, depression

## Abstract

**Background:**

As the number of adults who identify as autistic increases, it is vital to understand factors that improve autonomy and achievement of a good life for autistic adults. Postsecondary education and employment may contribute to higher levels of independence but may also add stress and increase mental health concerns. This study aimed to explore interactions of mental health concerns with adaptive daily living (ADL) outcomes, defined for this study as postsecondary education and employment outcomes, in verbally fluent autistic adults.

**Methods:**

We surveyed 140 adults with confirmed (*n* = 114) or self-reported (*n* = 26) autism diagnosis regarding autism traits, camouflaging traits, and mental health concerns including anxiety, depression, and intolerance of uncertainty. At the same time, we asked for detailed reports of postsecondary educational and employment activities for data needed to rate an adapted version of the Vocational Index and also for report of daily activities using the WHO Disability Assessment Schedule 2.0 scale. A structural equation model tested hypothesized relationships among these factors.

**Results:**

Autistic traits and camouflaging traits did not directly predict ADL outcomes. However, the mental health latent variable was a strong direct predictor of ADL outcomes in that worse mental health predicted better ADL; mental health also significantly mediated the relationships between autism traits and ADL and camouflaging traits and ADL.

**Conclusions:**

This sample self-reported substantial education and work experience, though not always at levels high enough to support independence. Nonetheless, overall success in adaptive daily activities remains a significant challenge. Importantly, success in school and work was predicted by poor mental health, an indicator of the high cost of fitting in with neurotypical expectations that highlights the need for neurotypical systems to better understand and accommodate unique strengths and support needs to facilitate success *and* well-being for autistic adults.

## Abstract (plain language summary)

Community Brief

Why is this topic important?

The number of adults who identify as autistic is growing quickly, so it is essential to understand what factors contribute to success and a good life.

There are growing numbers of programs to support autistic adults in post-secondary education and training for better jobs. However, many autistic adults report that these programs do not meet their needs.

What is the purpose of this article?

Our aim was to explore how mental health concerns, which are a common challenge for many autistic adults, affect everyday adult living activities (ADLs) including school, work, and community. We want to understand how much activities of daily living (ADLs) are related to camouflaging of autism traits and mental health.

What did the researchers do?

We surveyed 140 adults with confirmed or self-reported autism diagnosis about their ADLs including work, education, and wellbeing. We also asked questions about mental health and masking/camouflaging.

What were the results and conclusions of the study?

For many people who do not identify as autistic, ADLs are associated with better mental health. But for autistic adults in our survey, doing better at school and work was related to worse mental health. Our findings show that trying to “fit in” to succeed at school, work, and other daily activities might have a big cost for mental health for autistic people.

What is new or controversial about these findings?

Many studies with neurotypical people find that more success at work and school is related to better mental health. We found the opposite. This highlights a difficult balance that many autistic people have to navigate: be myself or be what others expect? There are ups and downs to both.

What are potential weaknesses in the study?

Our survey was conducted during the pandemic so extra stress people were feeling may have impacted the findings. Our sample size was on the smaller end of what is preferable for the statistics that we used. We did not use an adequate measure for describing gender.

How will these recommendations help autistic adults now or in the future?

We suggest that school and work programs must be designed with input from autistic people to identify successful and supportive polices. Education for human resources and education personnel is essential to dispel myths and open opportunities. We believe that many accommodations for supporting autistic people, such as installing more comfortable lighting, will be good for everyone. We hope that this understanding helps families, researchers, practitioners, and communities to actively, positively support and respect the strengths that autistic people bring to home, school, and work so that autistic people can feel more comfortable just being themselves and not trying to be someone else just for the sake of fitting in.

## Introduction

As the number of adults who identify as autistic increases rapidly, understanding factors that contribute to autonomy and achievement of a good life for autistic adults is becoming increasingly important.^1–5^ Research in this area is constrained by a paucity of measures of quality of life that have been developed and validated specifically for autistic adults.^1,6–8^ Existing studies using nonoptimal measures conclude that autistic adults report more challenges and poorer well-being compared with peers for many measures of independent, adaptive daily living (ADL), including measures of social interactions, self-care, physical health, mobility, leisure, and community participation.^9–11^ Importantly, these struggles in ADL may be bi-directionally associated with mental health challenges for autistic adults.^4,12–16^

Two important elements of ADL that are vital for this study include postsecondary educational experience^17–21^ and the quality and consistency of employment.^22–25^ For example, in general population studies, better long-term mental health is associated with both higher education^26–28^ and steady employment.^29–31^ However, relationships between work, education, and mental health also interact with individual and systemic variables that mediate both directionality and strength of impact; for example, work-related concerns related to low perceived control, relational injustice, stressful roles, or bullying; and varied challenges in education settings such as personal needs insecurity or strong institutional pressures to achieve without concomitant personal support increase mental health burdens for many people.^32–36^

There is less research to date about mechanistic relationships among employment, education, and mental health in autistic adults. A large survey study of 370 autistic adults by Mason et al.^22^ reported that being employed is a positive predictor of mental health, and a longitudinal comparison of surveys for 144 autistic adults, taken just before the onset of the COVID-19 pandemic and about 10 weeks after the COVID-related shutdown in the United States, showed that job loss or job reduction was associated with higher levels of depression even after accounting for baseline depression.^24^ And while autistic university students in multiple countries enjoy the freedom and autonomy they enjoy, they simultaneously struggle to manage multiple academic and living responsibilities, and report generally poor support especially for non-academic aspects of their experience.^17–21^ Overall, autistic adults report many more mental health challenges than their peers in higher education or employment settings, including more loneliness, depression, anxiety, and significantly increased risk for suicidal thoughts and behaviors.^14,37–42^ However, the nature of these interactions is not understood.

This study is part of our larger research trajectory to evaluate a range of variables that contribute to the well-being of autistic adults, including factors that increase opportunities for success as well as factors that decrease opportunities for success. Specifically, this study arises from perspectives that we hear frequently from autistic adults in clinical settings, as well as in our own and others’ research: masking or camouflaging autistic traits feels necessary to succeed in many work and school settings,^43–45^ but the effort to camouflage is so exhausting that there is little remaining time or energy. This leads other aspects of life to suffer substantially, including significant detriment to mental health.^43,46–49^ In our experience, many autistic adults wonder if the trade-off is worthwhile but feel that they have little option but to persevere trying to fit into normative societal expectations.

Research on autism and employment has historically focused on supported employment for adults with higher levels of support needs,^50–53^ often completed by carers or other informants. As part of an emerging emphasis in autism research on adding outcomes related to competitive, independent employment,^54^ we sampled only verbally fluent adults in this study.

Autistic traits refer to differences in social communication, preferences for sameness, and atypical sensory experience that reflect diagnostic criteria for autism; no single measure has been identified that adequately captures autistic traits in one place, especially for adults.^55–57^ Recent research efforts have shown repeatedly that the presentation of autism traits in females differs from their male counterparts, in part due to the frequency and intensity of camouflaging that autistic females portray and more use of socially oriented language and behavior in females.^31,49,58–61^ Mental health research in autistic adults consistently shows higher levels of challenge for females compared with males, which may arise partly from increased camouflaging.^46–48,62,63^ The most commonly used tools for autism diagnosis were constructed around the presentation of autistic traits found in males and may not adequately capture what it means to be female and autistic.^64^ This poses challenges for diagnosing and understanding autism in females, but as autism researchers and clinical practitioners come to recognize the number of girls and women who have historically been “missed” for diagnosis,^65–67^ it becomes imperative to explore how functional mechanisms and life experience may differ across biological sex.

Our aim was to explore this camouflaging conundrum in 140 verbally fluent adults who have confirmed (*n* = 114) or self-reported (*n* = 26) diagnoses of autism. We measured two latent outcome variables:

1. ADL, measured for this study using the WHO Disability Assessment Schedule 2.0 (WHODAS 2.0)^68^ and a version of the Vocational Index (VI)^69^ that measures educational and employment status in autistic adults, which we adapted to rate based on self-report survey data rather than a carer-reported measure.
2. Mental health concerns including self-report measures of anxiety, depression, and intolerance of uncertainty.

We explored how autism traits and camouflaging of autism traits impacted ADL and mental health outcomes. We additionally tested the role of mental health concerns as a possible mediator of the relationships between ADL and autism or camouflaging traits. We finally analyzed biological sex as a direct predictor of ADL to explore possible sex-specific contributions.

Our specific hypotheses were as follows:

1. ADL scores will be separately predicted by autism traits and camouflaging traits. We specifically hypothesized that higher autism traits would decrease ADL, while camouflaging traits would increase ADL.
2. Mental health concerns will be separately predicted by autism traits and camouflaging traits. We hypothesized that higher autism traits and camouflaging traits would increase mental health concerns.
3. Mental health concerns will mediate the relationships between autism traits and ADL and camouflaging traits with ADL. Specifically, because increased autism and camouflaging traits predict for increased mental health concerns, mediation should reflect decreased ADL.

## Methods

### Participants

The study was approved by the Brigham Young University Institutional Review Board in accordance with the Declaration of Helsinki. Inclusion criteria were age 18 years or older, native English speaker, and self-reported autism diagnosis. Self-reported diagnosis of intellectual disability, any personality disorder, a schizophrenia spectrum disorder, or bipolar disorder was excluded *a priori* in order to focus on the cluster of autism, anxiety, and depression. We recruited verbally fluent (self-reported) adults because this segment has been historically overlooked in autism employment research. Participants were recruited via existing research databases, invitations to advocacy organizations, and social media. Target sample size was based on a previous study by Maisel et al. using structural equation modeling (SEM) as a primary analysis with three mediational models embedded in the SEM,^70^ which used the Autism Spectrum Quotient (AQ) and a *combined anxiety* score taken from the State-Trait Anxiety Inventory, Penn State Worry Questionnaire, and Fear of Negative Evaluation–Brief. Maisel et al. found the fit of the SEM model to be excellent χ2(13) = 13.89, *p* = 0.381; RMSEA = 0.021; CFI = 0.998; TLI = 0.997 with a combined sample of 151 autistic and non-autistic adults. *A priori* power analysis using the Soper method suggested a minimum sample size of 90 participants for our structural model to achieve an effect size of 0.3.^71^

Our initial sample included 172 participants who started the survey. Of these, 24 participants did not get far enough into the survey to answer our trait questionnaires and outcome questions and were excluded. Eight participants not living in the United States were excluded from data analysis. Our final sample included a total of 140 participants (males = 71, females = 68, and one who did not respond to the question on sex-at-birth). Of these, 114 had a confirmed autism diagnosis through previous research or clinical contacts with the investigators or who provided evidence through diagnostic reports as part of the recruiting process; the remaining 26 were recruited via social media contacts and self-reported their diagnosis.

### Measures

Measures were completed online using Qualtrics software (Qualtrics Inc., Provo, UT, USA). To maintain overall brevity and usability for the study, we chose concise measures of each variable.

#### Demographic information

Participants provided information on their age, race, sex-at-birth, previous autism diagnosis, age at autism diagnosis, and any other neurodevelopmental or mental health conditions.

#### Individual traits

##### Autism Spectrum Quotient

The AQ^72^ is a 50-item self-report measure of autistic traits in adults with average to above average intelligence intended to capture the dimensions of autism phenotype in clinical and the general population. The AQ has previously been used for a study on employment outcomes for autistic adults and shows good test–retest reliability (*r* = 0.7).^73^

##### Camouflaging Autistic Traits Questionnaire

The Camouflaging Autistic Traits Questionnaire (CAT-Q)^74^ is a 25-item self-report measure of social camouflaging behaviors (i.e., strategies used to compensate for or mask autistic characteristics to facilitate social assimilation) intended for online administration. The CAT-Q measures both successful and failed camouflaging attempts based on intention.

Although there is not a clear cut-off score for CAT-Q, the mean score for autistic adults is 4.79. The measure’s internal consistency (α = 0.94) and test–retest reliability (*r* = 0.77) were good.

#### Adaptive daily living

##### WHO Disability Assessment Schedule 2.0

The WHODAS 2.0^68^ is a 36-item self-report measure of health and disability in adults across cultures. The WHODAS 2.0 is designed to measure an individual’s functioning in six major life domains including cognition, mobility, self-care, getting along, life activities, and participation. Burgeoning evidence for the use of the WHODAS 2.0 in adult autistic samples indicates high internal consistency of 0.934–0.95 and strong correlation of total score with domain scores (*r* = 0.66–0.84).^75,76^ Our analyses calculated the standardized score for interpretation of the total score; the WHODAS simple scoring method was used for the subscales of the WHODAS 2.0, which were included as indicators of the outcome latent variable.

##### Adapted (self-report) Vocational Index

Outcome for academic achievement and employment among autistic adults has been measured in various ways^19,77^ with no well-established measure of these outcomes to date. The VI^69^ was specifically created to be used by carers of autistic adults with moderate to high support needs. The VI consists of 11 statements indicating level of engagement in academic and employment activities based on work and educational settings and level of needed support (see Table 1). Each question of the VI is rated from 1 to 9.

**Table 1.** Comparison of Descriptive Data for Predictor and Outcome Variables by Sex at Birth

	Sex at birth				Comparison		
Measure	Female (*M*|*SD*)		Male (*M*|*SD*)		*t*	*p*	Effect
Predictor variables							
AQ total	34.57^a^	8.22	28.96^a^	6.81	4.38	<0.001	**F > M**
CAT-Q mean	4.96^a^	0.87	4.29	0.90	4.48	<0.001	**F > M**
Anxiety total	22.25^a^	6.21	20.44^a^	7.20	1.59	0.057	F = M
Depression total	22.26^a^	7.77	21.69	8.10	0.43	0.335	F = M
IUS-12 total	44.13^a^	9.34	39.11^a^	10.46	2.97	0.002	**F > M**
Outcome variables							
VI-Adapted	6.61	3.11	6.53	3.12	−0.14	0.443	F = M
WHODAS 2.0							
Total (standard)	81.91^a^	23.0	68.06^a^	21.95	3.63	<0.001	**F > M**
Cognition RAW	15.01^a^	4.36	13.15	4.40	2.50	0.007	**F > M**
Mobility RAW	9.06	4.31	7.93	3.43	1.71	<0.045	**F > M**
Self-care RAW	6.99	3.09	5.01	1.70	4.68	<0.001	**F > M**
Getting along RAW	13.04^a^	4.01	10.89	4.60	2.94	0.002	**F > M**
Life activities RAW	21.97^a^	7.52	17.72	7.36	3.37	<0.001	**F > M**
Participation RAW	20.75^a^	6.49	17.44	7.06	2.87	0.002	**F > M**

^a^ Above cut scores for that measure. Significant effects for sex are bolded.

Anxiety, PROMIS Anxiety 7a; AQ, Autism Quotient; CAT-Q, Camouflaging Autistic Traits Questionnaire; Depression, PROMIS Depression 8a; IUS-12, Intolerance of Uncertainty Scale-12; VI-Adapted, Vocational Index, adapted for researcher rating based on self-report survey of Educational and Vocational Activities; WHODAS 2.0, World Health Organization Disability Assessment Schedule 2.0 (higher scores indicate higher degree of struggle).

**Table 2.** Vocational Index Adapted for Research Rating Based on Self-Reported Activities

Score	Category	Frequency	Percentage of sample
9	Employment in the community *without* supports greater than 10 hours a week and/or postsecondary, *degree-seeking* educational program greater than 10 hours a week	61	44%
8	Postsecondary, *degree-seeking* education program or employment in the community *without* support—total activities 10 hours a week or less	16	11%
7	Employed in the community *with* supports greater than 10 hours a week. No time spent in sheltered settings	25	18%
6	Employed in community *with s*upports (no time spend in sheltered settings)—total activities 10 hours a week or less	1	<1%
5	Sheltered vocational setting and supported community employment—total activities greater than 10 hours a week	4	3%
4	Sheltered vocational setting and volunteering in the community—total activities greater than 10 hours a week and/or sheltered vocational setting (workshop or day activity center) with no community employment/volunteering—greater than 10 hours a week	2	1%
3	Sheltered vocational setting—total activities 10 hours a week or less	1	<1%
2	Volunteering with no other activities or postsecondary *non-degree-seeking* education with no other activities	3	2%
1	No vocational/education activities	27	19%

Ratings for this adapted measure come from the Educational and Vocational Activities Scale created for this study.

The parent-report VI has been used in studies looking at the longitudinal patterns of postsecondary and employment activities engagement.^3^ The VI provides a useful summary statement that integrates quantity of activity framed by level of independence. It has demonstrated good internal consistency (α = 0.92).^61^ Because we were investigating educational and employment experience of verbally fluent autistic adults, we modified the VI to be rated by researchers or clinicians based on answers compiled in an Academic Achievement and Employment Outcome Scale, including questions such as how many hours a week does the participant work, what kind of postsecondary education program they are enrolled in or graduated from, whether supports from family or the job are needed, and so forth; the complete scales are presented in the Supplementary Data. We administered the Academic Achievement and Employment Outcome Scales via Qualtrics and researcher S.Y.P. assigned the rating for each VI item based on that information.

#### Mental health concerns

##### Patient-Reported Outcomes Measurement Information System Depression (8a) and Anxiety (7a) Scales

Fixed-length short-form self-report measures have been developed as part of the Patient-Reported Outcomes Measurement Information System® and are part of the research battery supported by the National Institutes of Health. The measures ask individuals to rate how often they’ve felt depression symptoms (e.g., “I felt worthless”; “I felt that I had nothing to look forward to”) and anxiety symptoms (e.g., “I felt fearful”; “I felt anxious”) in the past 7 days. Raw scores are computed by summing the scores and converting the raw scores to T-scores using the conversion tables for each of the measures. Both measures show good reliability and validity with reference to other depression and anxiety scales.^78–80^ Sensitivity has been strong in multiple studies, though specificity for specific anxiety disorders lags behind.

##### Intolerance of Uncertainty Scale-12

The Intolerance of Uncertainty Scale-12 (IUS-12)^81^ is a 12-item self-report measure regarding prospective anxiety about the unknown (e.g., “Unforeseen events upset me greatly”) and inhibitory anxiety (e.g., “Uncertainty keeps me from living a full life”). The IUS-12 has excellent internal consistency (α = 0.93), good criterion validity (*r* = 0.98), and good test–retest reliability (*r* = 0.77).^82^ The IUS-12 has been successfully used to show an association between intolerance of uncertainty and anxiety in adolescents and adults diagnosed with autism.^70,83^

#### Pilot survey review by autistic advisors

A pilot survey was completed by four autistic adults (three from USA; one from Australia) who provided commentary on their experience. This feedback led to clarification of survey instructions and specific questions and to shortening the length where possible while maintaining standardized questionnaires in their original form, to improve understanding and to prevent fatigue.

### Procedures

All participants provided consent via a Qualtrics-based form before they completed a screening survey. Individuals who met inclusion criteria and indicated interest on the screening survey received additional instructions for completing the study and links to all measures via email. Upon completion of all measures, participants with confirmed diagnosis of autism spectrum disorder and who were invited directly by the researchers (*n* = 114) received compensation for their time the equivalent of USD $10 in the form of an Amazon gift card.

Procedures for participants recruited via social media were modified after an initial attempt to recruit via social media was hijacked by unscrupulous persons seeking payment using bots^84,85^ or other nonlegitimate responses. Intense scrutiny of these responses suggested none were valid and all of these results were discarded. Procedures for the subsequent social media included:

• Possible compensation via a drawing for an Amazon gift card equivalent of USD
• $40 with a one in four chance of winning
• A simple quiz using questions to establish proficiency in English and discourage automated responding
• Attention checks throughout the survey to evaluate legitimate human responding

The second round of data from social media invitees (*n* = 26) was thoroughly vetted and determined to be valid.

### Data analysis

Preliminary to the main analysis, data were prepared by reverse-coding items as needed and calculating the total raw scores for each of the measures across all participants, with the exception of the CAT-Q, which used mean scores, and the WHODAS 2.0 total score, which utilizes a formula provided by the World Health Organization.^86^ The VI scores were calculated by investigators based on individual responses to the Academic Achievement and Employment Scale.

We chose to use structural equation modeling to test the theoretical models based on explicitly stated concepts: specifically, to take into account the dimensional nature of measurements both of autism traits and camouflaging traits^49,70,87–89^ as they relate to the ADL and mental health concerns outcome variables. Mediational analyses allow for evaluation of the mechanisms through which these traits affect the outcome variables. We thus created an SEM model with the latent factors for ADL and mental health concerns. Mediational relationships were analyzed both within the SEM model, which allows for testing the entire model, and the mediational effects in a single analysis. Sex was included in the SEM model as a predictor. All of the analyses were conducted using STATA 16.0.

## Results

### Confirmed versus self-reported participants

There was no difference in AQ total scores between participants with confirmed (mean [*M*] = 31.72, standard deviation [*SD*] = 8.24) versus self-reported autism diagnoses (*M* = 32.04, *SD* = 7.42), *t(138)* = 0.181, *p* = 0.856. The two groups were combined for all subsequent analyses.

### Demographics

The total sample included 68 participants who reported a female sex at birth, 71 males, and 1 who did not answer. To enhance privacy, we asked for age in ranges rather than specific years. There was a wide range of ages, though nearly half of the sample were young adults. The total in each age category included: ages 18–24, *n* = 62, 44%; 25–30, *n* = 44, 31%; 31–40, *n* = 21, 15%; 41–50, *n* = 10, 7%; 50+, *n* = 3, 2%. Ninety-nine participants (71%) reported being single and never married, while 31 were married (22%), 6 were divorced (4%), and 4 did not report. The age of autism diagnosis varied across the whole range including a diagnosis under age 6 (*n* = 25, 18%), ages 6–12 (*n* = 19, 14%), ages 13–17 (*n* = 22, 16%), ages 18–24 (*n* = 37, 26%), ages 25–30 (*n*= 14, 10%), ages 31–50 (*n* = 18, 13%), and one participant diagnosed over age 50 (1%).

### Descriptive data

#### Individual traits

Table 1 shows descriptive data separately for females and males, including how many scores are above cut scores for that measure and a comparison of sex differences for each measure. Results indicate an expected high level of autism traits in this sample, as mean scores for both females and males were above the screening cut score of 26 on the AQ.^72^ Self-reported camouflaging traits were also high, with both females and males close to the mean score of 4.79 that previous research suggests is a concern for autistic samples.^90^ Women scored higher than men on both scales.

#### Adaptive daily living

WHODAS 2.0 total scores indicate significant struggles in everyday life for this sample. Mean scores for both women and men fell in the range of *severe difficulty* and women report markedly more struggles than men. The cognition, getting along, life activities, and participation domain scores were all in the *severe* range of difficulty, while the mobility and self-care domains were in the *moderate* range of difficulty.

Questions and results for the adapted VI are summarized in Table 2. Overall, a slim majority (55%) of this select sample of verbally fluent autistic adults is engaged in completely independent postsecondary education pursuit and/or employment (scores of 8 or 9 on the adapted VI). The next group (scores of 6 or 7) reports reliance on some supports—for many of them, reliance on family members for transportation. Nearly 1/5 of our sample (*n* = 27) reported no ongoing postsecondary education or employment activities.

#### Mental health concerns

Reports of mental health concerns were high, with mean scores above cut score for clinical concern of 20 for the anxiety scale^91^ for both women and men, and similar concern for depression, with mean scores for women just above and men just below the cut score of 22.^92^ Both women and men were substantially above the cut score for clinical concern of 28 on the IUS-12,^82^ indicating marked difficulty in situations that are uncertain or ambiguous.

### Correlational relationships

As shown in Table 3, WHODAS 2.0 total scores were significantly negatively correlated with the VI scores and significantly positively correlated with all trait measures; there was no difference in strength of correlation between anxiety and depression measures. VI scores were not significantly correlated with any trait variable. Mental health traits were significantly correlated with all outcome and other trait scores.

**Table 3.** Correlations of WHODAS 2.0 Total Score with VI Outcomes, Mental Health, and Autism Traits

	WHODAS 2.0 total *r =*
VI-Adapted	−0.248^*^
Anxiety total	0.657^**^
Depression total	0.624^**^
IUS-12 total	0.549^**^
AQ total	0.450^**^
CAT-Q mean	0.442^**^

^*^ *p* < 0.05.

^**^ *p* < 0.01.

### Structural equation model

**FIG. 1.**
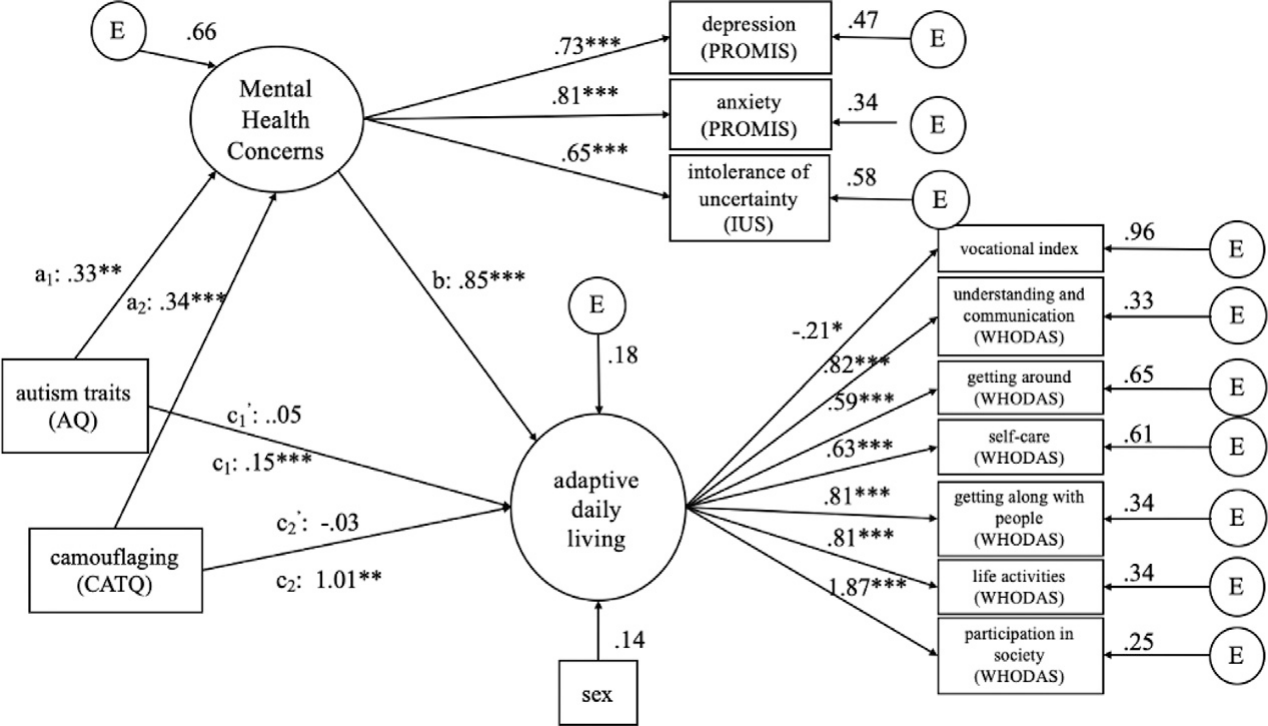
Structural equation model indicating tests of direct relationship and mediation of mental health concerns on educational and employment outcomes. All values are standardized beta coefficients. The arrows from AQ to Outcome and CAT-Q to Outcome show both direct effects (c_1_’ and c_2_’) and total effects (c_1_ and c_2_). AQ, Autism Spectrum Quotient; CAT-Q, Camouflaging Autistic Traits Questionnaire.

The full SEM model is shown in Figure 1. Contrary to Hypothesis 1, neither autism traits (AQ scores, c_1_’) or camouflaging traits (c_2_’) directly predicted the ADL latent variable. Instead, ADL was predicted by mental health concerns (β = 0.85, *p* < 0.001) in a different direction than predicted: *increased* mental health concerns were associated with *increased* ADL success.

Hypothesis 2 received strong support as both autism (β = 0.33, *p* < 0.01) and camouflaging traits (β = 0.34, *p* = 0.01) predicted increased mental health concerns on a latent variable including anxiety, depression, and intolerance of uncertainty. As suggested by Hypothesis 3, mental health scores mediated the relationship between autism traits and camouflaging traits with the ADL variable, but again in an opposite direction than predicted (c_1_ = 0.15, *p* < 0.001; c_2_ = 0.101, *p* < 0.01).

## Discussion

### The costs of camouflaging

In self-report questionnaires completed by 140 autistic adults (116 clinically, confirmed, 26 self-reported), we measured ADL outcomes defined by six life domains measured by the WHODAS 2.0, as well as self-reported activities in postsecondary education and employment. We also defined a latent mental health concerns variable using measures of depression, anxiety, and intolerance of uncertainty. In non-autistic populations, better mental health most often predicts better performance in school and job settings.^26–31^

However, in this study of autistic adults, better performance in school and job setting was strongly predicted by *worse* mental health. Additionally, we found that mental health significantly mediated two pairs of relationships between autism traits and ADL and camouflaging traits and ADL. These data highlight the important interconnected relationships between expected everyday performance including education and employment, with ongoing mental health status, and with efforts to conceal autistic traits at school and work.

Knowing that both autism traits *and* masking of autism traits predict worse mental health puts autistic adults in a frustrating bind: to mask or not to mask? Hypothesis 3 had predicted that mental health would mediate the relationship between autism traits and ADL outcomes, but results were in an unexpected direction. Consultation with our autistic research advisory group as well as our autistic clients has emphasized the take-away interpretation that high costs of performing in public, in order to succeed within a neurotypical framework, are often followed by feelings of anxiety and exhaustion. Masking is necessary to succeed but ultimately disrupts mental well-being. Although autistic adults already experience increased vulnerability for mental health challenges related to biological, cognitive, and systemic societal factors,^15,93,94^ requirements to mask or camouflage autism traits in order to be accepted can add to the mental health burden.^48,49,58,90^ A recent survey^95^ of autistic adults in the UK who are employed or seeking employment likewise highlighted the notion of “workplace masking as a perceived necessity”: participants wished it were not necessary but recognize that at this time in society, “fitting in” is often a requirement for employment success.

### Indicators of success

There are several positive findings from this study. According to self-report data, nearly three-fourth of the sample is engaged in regular (10 hours/week +) postsecondary and/or employment activity across a broad age range of young- to middle-aged adults. Many are enrolled in degree-seeking programs. Among those receiving some support, such as transportation from families, many report enjoying independence in other areas of their lives. In the context of increasing education and employment opportunities in wider areas of skill and interest,^96,97^ opportunities for meaningful, successful outcomes may be better than ever before.

### Indicators of challenges

It is vital to adopt a strengths-based approach to achieve more successful ADLs for educational and employment experiences for autistic adults.^98–100^ An important step in this process is recognizing where the challenges lie in order to target them directly. This study highlighted a number of such challenges. Overall scores for ADL measured by the WHODAS 2.0 show that four domains are in the *severe* range of concern and the other two domains are in the *moderate* range of concern. Compared with peers, this sample of autistic adults reports significant difficulty and distress in many areas of everyday life such as making friends, taking care of house responsibilities, and getting around.

A close look at data in the Educational and Employment Activities scale that was used to rate the VI shows that autistic adults are taking a long time to complete their degree programs, thus delaying entry into the work force. Many in this sample who are employed are working too few hours to reasonably support independent living. An intersection of delayed graduation rates, lower hours of employment, and being underpaid is likely to pose as a barrier to greater employment opportunities.

### Suggestions for improvement

#### Developing programs

There are now many programs embedded in postsecondary education settings that utilize some combination of trained counselors and peer mentors to support autistic students in connecting to campus-based services, coaching on organizational and life skills, and mental health and wellbeing.^17,97,101–105^ Wolpe^101^ summarizes benefits these programs can provide, including (1) opportunities to connect with other students with similar interests and (2) finding understanding with other autistic peers. Nonetheless, many participants continue to report that their programs do not actually meet their needs across multiple areas, for example, for participating in the social components of university life, developing strategies for academic success, support for managing their own autism identity within the context of discrimination by others, and additional mental health needs.^19,20,95,101,106^

Similar to new initiatives in postsecondary education, there are marked increases in efforts to expand employment access for autistic adults that capture diverse talents, interests, and abilities.^95,96,107–109^ Large and small employers are revisiting recruitment, hiring, and onboarding policies, and the U.S. Department of Labor has compiled a resource list that describes best practices and model programs.^96^ However, there is little quality research on how well these new programs are working so that companies that mean well may not actually be providing effective opportunities for success.^51,107^

#### Ideas from research

There is an urgent need to shift part of the responsibility for successful outcomes from the students and employees to teachers, mentors, and supervisors. For example, human resources departments and college counseling departments should deliberately undertake professional development workshops that dispel myths about autistic adults and encourage reasonable accommodations are likely to benefit everyone.^98,99,110^ Some examples include (1) providing education about the nature of disabilities including autism; (2) confronting beliefs, with data, that people with disabilities perform more poorly at their work and/or have more accidents and/or sick days; (3) demonstrating a strong return on investment from effective work and how providing accommodations requires minimal if any extra expenditure of resources; and (4) attending to environmental aspects of the workplace including sensory elements such as lighting and sound; for instance, changing out standard fluorescent lighting for LED lighting and/or lighting that can change levels throughout the day and/or increasing the color temperature positive impacts alertness, attitude, and energy level for children and adults generally with or without identified disability status.^111–113^

Just as importantly, there is need for specific supports at every stage of what Davies et al.^95^ refer to as the “life cycle employment” from knowing what jobs to look for, to skills needed to obtain the job, to knowing about how to get promoted or move into higher-skill jobs, to knowing how to transition out of the workplace at the right time. Specific target areas suggested by Coleman and Adams^114^ include roles for (1) job coaches to “pre-interview” with employers to review strengths and difficulties for the incoming employee; (2) training staff about autism expectations and decreasing bias; 3) providing workplace peer mentors (in addition to or replacing job coaches); and (4) encouraging effective transportation training and/or equipment such as public transportation, car/van pooling, support for bicycles if safe routes exist, and so forth. See Coleman and Adams^114^ and Davies et al.^95^ for additional ideas.

Nicholas and Klag^23^ emphasize the need for a more collective and comprehensive approach in addressing employment disparities that autistic adults experience. Programs geared toward increasing employment opportunities (increase in employment rates, ability to use skills, appropriate pay) need to focus on the overall goal of improving life rather than having an endpoint of merely obtaining a job. Further focus on long-term employment, development of strengths, and community-based programming can support ongoing employment. In fact, autistic adults who were enrolled in training programs that were designed to increase long-term employment skills, rather than short-term skills, produced better employment outcomes, and employers were more likely to view employing autistic adults as being positive. Furthermore, including coworkers led to positive learning and skills building for both autistic and non-autistic workers.

In both education and employment settings, there is urgent need for program designers to consult with and think about autistic people at all phases of development, who can provide specific guidance on elements that will actually work for them.^13,15,23,95,115^ For example, architects and university planners benefit from collaboration with autistic consultants and autism experts in the co-design of campus spaces for living, learning, and recreation that are sensory-friendly and allow for easier communication.^116–118^ Acknowledging that budget constraints and existing structural barriers may prevent a fully neuro-divergent acceptable space, there are many fixes that are reasonable and enhanced by co-production. de Vries^119^ argues that sensory-friendly office spaces (e.g., mitigating or removing fluorescent lights, perfume odors, and offensive noises) are an ethical and should be a legal imperative for employers. Pfeiffer et al. found that peer and management support, clarity about job roles, and physical comfort were positively associated with job satisfaction for autistic employees.^120^

These and other studies show that autistic students and employees know what works and what doesn’t work well; consulting autistic people themselves is essential to improving school and work settings that benefit all.^95,100^

### Strengths and limitations

The self-report study design allowed researchers to capture the firsthand experience of autistic adults who identified as verbally fluent and able to read and complete the online surveys.

The study is complicated by the timing of data collection. The data were collected from November 2020 to May 2021, a period during which intensive COVID-19 restrictions had been placed. Many reports found increased mental concerns during this time alongside some possible benefits^121–123^ may have been affected for better and worse, and current education and employment levels may have been atypical, although past events (including past jobs and past program graduations) were included in the ratings for the adapted VI.

Another limitation of the present study is that the only questions related to gender identity were about sex-at-birth with the options to respond as male, female, or intersex. This decision mirrors previous studies on similar topics (e.g., Lai & Szatmari, 2020). However, the authors acknowledge the higher rate of gender variance in autistic population and the importance of separately studying sex and gender.^124^ Recent work has highlighted the need for asking about both sex and gender in autism research.^125^

Adequate sample size for SEM analyses is affected by a host of factors, and there are no appropriate one-size-fits-all rules.^126^ Latent variables such as used here are stronger than single indicators. Our sample was smaller but broadly in line with other studies that have directly surveyed mental health variables in autistic adults (i.e., not large-scale databases or general population samples), including Maisel et al.^70^ (*n* = 151), Riedelbauch et al.^127^ (*n* = 188), and Edwards et al.^128^ (*n* = 224). Larger sample studies will provide more certainty about key relationships among life outcomes, camouflaging, and mental health.

We did not find sex-based differences in the pattern of relationships for our SEM model. However, the little research available suggests that many autism trait measures, including the AQ that we used, may be better suited or biased toward a male presentation of autism than that seen in females.^129–131^ Thus, a better profile of autistic traits in females may lead to different conclusions. A new measure created in Dutch^132^ specifically regarding the Autistic Women’s Experience should pave the way for similar measures in other languages and cultures.

## Conclusions

Many autistic adults nurture unique cognitive and organizational skills that can contribute to success in everyday living but also face unique barriers. The mediation analysis reported in this study indicates that engagement in daily tasks may come at a great cost to mental health. More efforts are important to identify the specific areas in need of improvements to lessen the employment and academic achievement gap for autistic adults.

## Authorship Confirmation Statement

S.Y.P. designed the study, was responsible for data collection, and wrote an initial draft of the paper. M.B. completed additional analyses and adapted the article to its current framework. M.S. provided funding for the study, access to the research database, and conceptual guidance throughout and wrote the final draft of the article. All authors have read and approved the final article. The article hasbeen submitted solely to *Autism in Adulthood*.
