# Supplementary material for: Mental Health as a Key Mediator for Outcomes in Postsecondary Education, Employment, and Everyday Living in Autistic Adults
Source: Autism Adulthood. 2025 Aug 11;7(4):479–92. doi: 10.1089/aut.2024.0122 (PMC12417845; doi:10.1089/aut.2024.0122)
Supplement: Supplementary file 1 [file aut.2024.0122_supplementary_data.docx]

**Supplemental Data: Educational and Vocational Activities Index**

*Used to Rate the Vocational Index - Adapted*

The following questions ask about job and education activities you participate in during the week on a regular basis. Most questions will start with a title followed by explanations and examples of what is meant by the question, then the question itself. Please read these carefully to best understand how to answer it for yourself. Just make your best guess for each question.

**Employment**

*How many jobs?*

*Explanation: some jobs are volunteer and some jobs get paid a salary or hourly wage. This question asks only about jobs that get paid, for example getting a paycheck every two weeks or every month. If you do consulting or piece work, so that you get paid only when you do a project, count that as one job.*

*You may have had other jobs in the past, but this question is just about jobs you are employed right now.*

How many jobs do you work at for pay right now?

a) None
b) One
c) Two
d) Three or more

Display logic: Job 1. Display as follows if “one” is chosen. If two or more chosen, change to “These are questions about your first job” and “second job” or “third job”

*Job type.*

*Explanation: Type what kind of job you do. You do not need to write who you work for, just the kind of company it is. Examples might include: food service, working in a shop, construction work, software engineer, graphic designer, and so forth.*

What kind of work do you do? {free response}

*Hours at job.*

*Explanation: sometimes the hours that people work vary from week to week. But on average, for example over the past four weeks, what is the usual number of hours that you work?*

About how many hours per week on average do you work at your current job?

a) Up to 10 hours/week
b) 11-20 hours/week
c) 21-30 hours/week
d) More than 30 hours/week

*Wages.*

*Explanation: people are paid in different ways. Many are paid hourly. Some are paid an annual salary rate. Please just fill in the amount you are paid and what is the scale (such as hourly or annual). If you do consultation or piece work estimate your average annual income. Also, please put the country and currency you are paid in. Examples might include: $10.50/hour in US dollars; or £28,000/year in British Pounds. If you prefer not to say just type “Choose not to say”*

What is your wage for this job? {free response}

*Job support or assistance.*

*Explanation: sometimes people have a job where they are provided with some additional assistance than what is usually found for that job. This might be a job coach or aide who comes around sometimes or all of the time to make sure things are going well for you, or someone you can contact specifically if you need help with your job. This is different from a boss or manager that everyone has. It might be someone who works for a school or an agency or a government office specially to help people keep their employment.*

Do you receive any additional job support or assistance, compared to usual for that job?

a) No
b) Yes
c) Not sure

*Family support.*

*Explanation: Some workers rely on family support for their employment. This may involve family members driving the worker to and from the job, or other responsibilities to help the worker be successful.*

How much do you rely on your family to be successful at your job?

a) I am completely independent in my job
b) I partially rely on support from my family to be successful in my job but could do it without them if needed
c) I could not do my job without family support

*Agency or workshop placement.*

*Explanation: some schools, institutions, agencies or sheltered workshops that are meant specially for adults with learning differences or with various kinds of disabilities will hire people to work at their center, or they help students or residents to get job placements at other places.*

Do you work for a school or agency or workshop that is meant especially for people with learning differences or any kind of disability?

a) No
b) Yes
c) Not sure

*Regular volunteer work.*

*Explanation: Some people work without pay to help others in their community, such as working for a library or church or any organization that works with unpaid volunteers to get things done.*

*Most people help somebody out once in a while, but this question is asking for regular volunteer work that may or may not be scheduled but happens most weeks.*

Do you regularly do unpaid volunteer work in your community, working most weeks?

a) No
b) Yes
c) Not sure

Display logic: If yes

*Hours volunteering.*

*Explanation: sometimes the hours that people volunteer vary from week to week. But on average, for example over the past four weeks, what is the usual number of hours that you volunteer?*

About how many hours per week on average do you work at your volunteer job?

a) Up to 10 hours/week
b) More than 10 hours/week

Display logic: if “None” for number of jobs

Are you currently looking for a job?

a) Yes
b) No
c) A little bit of both
d) Choose not to say

Which of the following best describes the reason you are not working at present?

a) Health reasons
b) Termination from the previous job
c) Personal decision (for example: choosing to leave the previous job, choosing to not work)
d) Other reason
e) Choose not to say

**Post-Secondary Education (also called Tertiary Education)**

*Current education program.*

*Explanation: different countries have different educational systems but most make a distinction between regular secondary education (like a high school) and post-secondary or tertiary education (after high school). Examples of post-secondary education might include a university (or college in the U.S.) or technical school that people attend after their regular schooling.*

Are you *currently enrolled* in a post-secondary education program?

a) Yes
b) No
c) Not sure

Display logic: if yes:

*Educational program goals.*

*Explanation: some post-secondary programs are meant to end with a degree, such as an Associate’s degree or Bachelor’s degree or higher level of degree.* *This is different than training programs that end with a certificate or advanced diploma.*

What is the end goal of your current educational program?

a) Degree (such as a Bachelor’s degree)
b) Certificate (such as for a specific training program)
c) Other type of goal
d) Not sure

*Educational assistance.*

*Explanation: some post-secondary students receive accommodations or assistance to support their learning and living or special educational needs. Examples might include extra time for exams or leniency for assignment deadlines. Often such assistance is coordinated by a university accessibility center or similar office.*

Do you receive any extra educational assistance for your current program?

a) No
b) Yes
c) Not sure

Display logic: if no to current enrollment:

Have you ever attended a post-secondary or tertiary education program?

a) Yes
b) No
c) Not sure
d) Still too young (such as still in high school)

Display logic: If yes to ever attended:

*Educational Program Goals*

*Explanation: some post-secondary programs are meant to end with a degree, such as an Associate’s degree or Bachelor’s degree or higher level of degree.* *This is different than training programs that end with a certificate or advanced diploma.*

What was the end goal of your completed educational program?

a) Degree (such as a Bachelor’s degree)
b) Certificate (such as for a specific training program)
c) Other goal
d) Not sure

*Educational assistance.*

*Explanation: some post-secondary students receive accommodations or assistance to support their learning and living or special educational needs. Examples might include extra time for exams or leniency for assignment deadlines. Often such assistance is coordinated by a university accessibility center or similar office.*

Did you receive any extra educational assistance for your program?

a) No
b) Yes
c) Not sure

Did you complete your previous program where you received a degree or certificate, that is, did you get to the end of the program successfully?

a) Yes
b) No
c) Not sure
